# Insect immune systems: same same but different but still same

**DOI:** 10.3389/fimmu.2026.1884037

**Published:** 2026-08-13

**Authors:** Victor Cardoso-Jaime, Krystal Maya-Maldonado, Sydney Powell, Chinmay V. Tikhe

**Affiliations:** 1 Colegio de Ciencias y Humanidades, Academia de Nutrición y Salud, Universidad Autónoma de la Ciudad de México, Plantel Casa Libertad, Mexico City, Mexico; 2 Biochemistry Department, Center for Research and Advanced Studies, Mexico City, Mexico; 3 Department of Biology, Ball State University, Muncie, IN, United States

**Keywords:** immune system, insects, IMD pathway, TOLL pathway, JAK/STAT pathway, RNAi pathway, mosquito, drosophila

## Abstract

Insects are the most diverse group of animals in nature, occupying nearly every ecological niche and playing central roles as pollinators, pests, and disease vectors. Despite this vast diversity, insects rely on a set of conserved yet evolutionarily adaptable immune pathways to defend against pathogens. Early studies in insect immunity have laid the foundation for human immunology, and recent advances in genomic and transgenic technologies have renewed interest in understanding how immune responses vary across insect orders. Insects are highly diverse in their immune systems; each species has unique immune responses that help fight infections from specific pathogens. Nevertheless, they share multiple aspects of recognition, regulation, and effector mechanisms. This review focuses on current knowledge of the immune systems of major insect lineages to highlight both shared signaling pathways, immune cells, and humoral factors, as well as lineage-specific responses that reflect distinct ecological pressures that have shaped the host-microbe interactions. Comparing different insect species and orders not only provides insights into the evolutionary divergences and convergences of immune system features but also offers complementary knowledge among species within the same order, helping fill existing gaps. Understanding these evolutionary patterns not only deepens our understanding of insect immunity but also informs the development of transgenic strategies to disrupt pathogen transmission in key vector species.

## 1 Introduction

From pollinators to agricultural pests, and from medically important vectors to food sources, insects have a major impact on all aspects of life. Insects occupy nearly all ecological niches on the planet. This results in a stunning diversity of morphological and physiological adaptations. The smallest known insect is only a few micrometers long, while the longest is up to 60 cm. There are close to a million described insect species. The total estimated number ranges from 2 to 30 million. Insects represent one of the earliest animal lineages on Earth. The fossil record indicates their origin about 400 million years ago (1). Insects have a remarkable ability to adapt to diverse environmental conditions, including temperature, light, and other abiotic factors (2). Because of their adaptability to various niches, insects constantly interact with other organisms. These include plants, animals, and especially microbes such as bacteria, fungi, and viruses.

Insect-microbe interactions can be either harmful or beneficial (3, 4). Because of these interactions, insect immune systems have evolved and diversified to either eliminate or tolerate certain microbial associations (5). Although the degree and specificity of the immune response against a particular microbe are unique for an individual insect species, the core immune pathways are highly conserved across insect orders. The consensus in the field is that the genes involved in core signaling pathways remain highly conserved due to biological constraints. On the other hand, genes that interact directly with pathogens evolve rapidly via duplications, diversification, or loss of function. The most conserved immune pathways in insects are Toll, IMD (Immune Deficiency), JAK/STAT (Janus kinases/signal transducers and activators of transcription), and RNAi (RNA interference).

Understanding how the immune response regulates insect-microbial interactions is particularly important because many insect species act as vectors of plant, livestock, and human pathogens, posing major public health concerns and substantial economic losses (6–8). The transmission of vector-borne diseases largely depends on interactions between the insect’s immune system and the pathogens it transmits (9). Another reason to study the insect immune system is its role in determining the effectiveness of microbial biopesticides, which are widely used to control insect pests (10–12). The success of microbial insecticides depends on the insect’s immune system’s ability to defend against these pathogens. In recent years, several emerging novel strategies for insect population control involve manipulating insect immune responses to block infection or pathogen transmission, as well as directly targeting essential genes using RNAi-based silencing approaches (13, 14). Understanding insect immunity is also important for protecting beneficial insects, such as bees and other pollinators (15). Studying how pollinators respond to pathogens and parasites, particularly under global environmental stressors such as climate change, can help develop better strategies to improve their health and survival. Studies on insect immunity have also helped scientists better understand many basic principles of the immune system in humans and other animals, and they continue to do so today (16).

Insect immune system is a complex process involving Pattern Recognition Receptors (PRRs), signaling molecules, transcription factors, effectors such as antimicrobial peptides, lysozymes, prophenoloxidase system, and others. Additionally, cellular immune response involves the participation of specialized immune cells called hemocytes, which eliminate pathogens by phagocytosis, nodulation, and encapsulation. Although all these processes are important to correctly trigger an immune response, the immune pathways are the master regulators of the immune system (17). Here, we present a birds-eye overview of divergent and convergent immune pathways across all insect orders described to date. This comparative analysis highlights similarities and differences among immune pathways, identifies conserved mechanisms, and clarifies knowledge gaps in insect orders whose immune systems remain poorly characterized.

## 2 JAK/STAT pathway

The Janus kinase-signal transducer and activator of transcription (JAK/STAT) pathway is one of the most evolutionarily conserved signaling pathways across living organisms, including insects (18). The JAK/STAT pathway was initially described in *Drosophila melanogaster* as a key regulator of development (19). About a decade later, it was also identified as involved in the antiviral immune response (20); in fact, for *Drosophila* and mosquitoes, the JAK/STAT is a major antiviral pathway (21–23).

**Figure 1:**
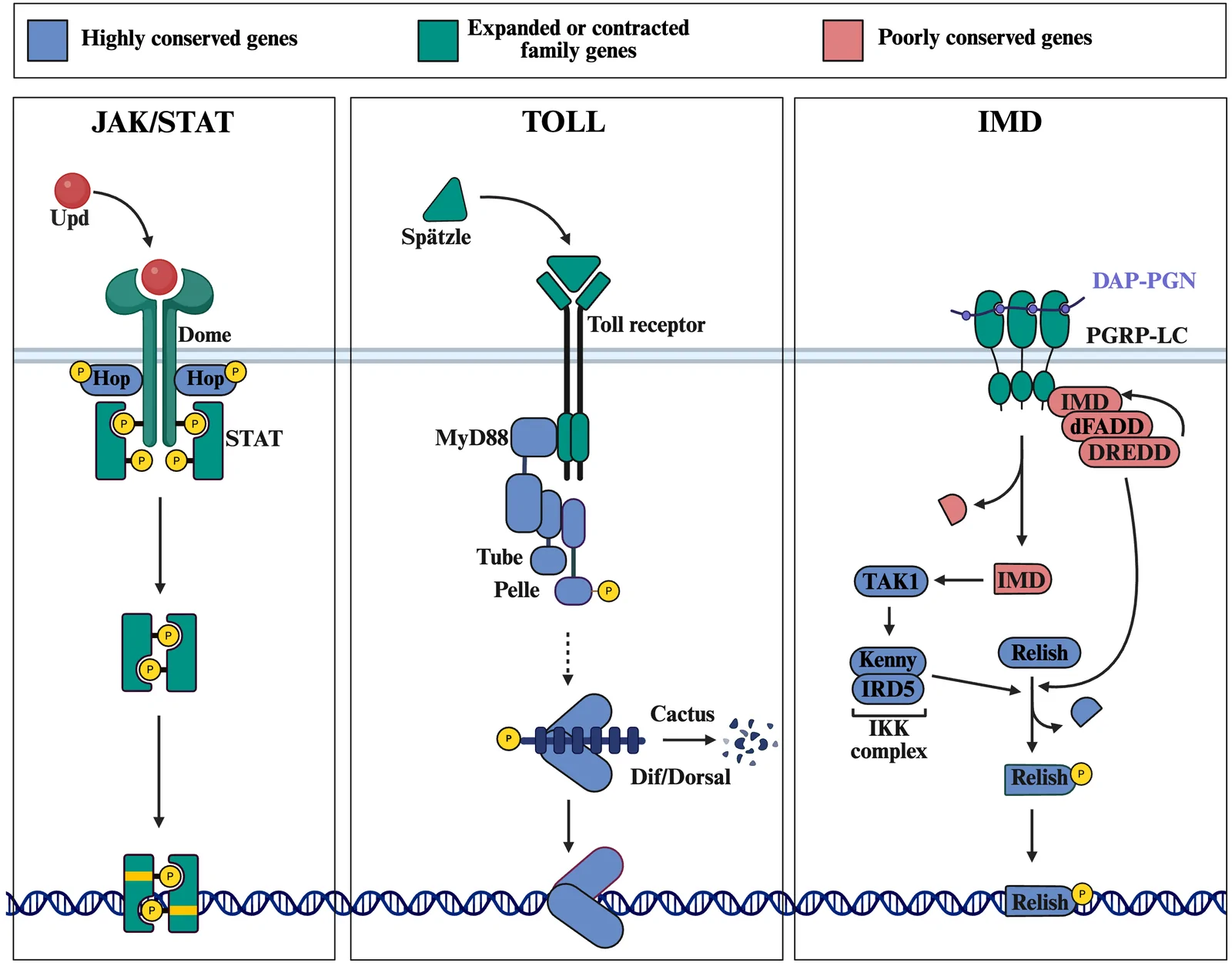
General overview of three major innate immune pathways in insects across different insect orders. The schematic illustrates the conserved core components and signaling mechanisms of the JAK/STAT, Toll and IMD pathways involved in insect immunity. The figure represents a generalized model compiled from currently available evidence across insects and may not fully reflect pathway composition or regulation in all insect orders. Created in BioRender. Maya-maldonado, K. (2026) https://BioRender.com/coe2p1a.

In *D. melanogaster* (Diptera), the JAK/STAT pathway is well characterized and has been widely used as a reference for studying this pathway in other insect orders. In this species, as in most insects, the core components of the JAK/STAT pathway include the ligands/cytokines Unpaired (Upd1, Upd2, and Upd3), the receptor Domeless (Dome), the Janus kinase Hopscotch (Hop), and the transcription factor STAT92E (24). Additional regulators, such as the suppressor of cytokine signaling (SOCS) and the protein inhibitor of activated STAT (PIAS), play an important role, but are not considered core components of the pathway (24). The pathway activation begins when the ligands (Upds in *D. melanogaster*) bind to the receptor Dome, which activates the Hop proteins that interact with the cytoplasmic portion of Dome. Activated Hop phosphorylates the Dome receptors; such phosphorylations serve as a docking site for the STAT dimer. STAT bound to the receptor is phosphorylated and then activated. The activated STAT dimer is translocated into the nucleus, where it serves as a transcriptional factor (24, 25) (**Figure 1**). Although this pathway is present in all insect orders in which immune pathways have been characterized, it is likely conserved in understudied orders. To date, the most extensively studied groups include Diptera, Lepidoptera, Hemiptera, Hymenoptera, Coleoptera, and Thysanoptera.

Interestingly, all core components are highly conserved except for UPD (**Figure 1**). For example, in *Drosophila* (Diptera), there are three Upd genes (Upd1–3) (26, 27). In the mosquito *Anopheles gambiae* (Diptera), only Upd3 has been described (28, 29). In other mosquitoes, such as *Aedes aegypti* and *Culex quinquefasciatus*, Upd has not been described. However, they express Vago, which in *Cx. quinquefasciatus* is a secreted protein that activates the JAK/STAT pathway independently of Dome and restricts viral infections (23, 30). Beyond Drosophila and mosquitoes, the pathway has been examined in several Dipteran species, including *Musca domestica*, *Wohlfahrtia magnifica* (a myiasis-causing flesh fly), and *Lutzomyia longipalpis* (31– 32). In almost all of these species, a single copy of Dome, Hop, and STAT has been reported. Some exceptions are several mosquito species that have STAT-A and STAT-B genes (32, 33). In Dipteran insects, the JAK/STAT pathway mediates immune responses against bacteria, viruses, parasites, and parasitoid wasps (19–22, 30, 31, 34–40).

**Figure 2:**
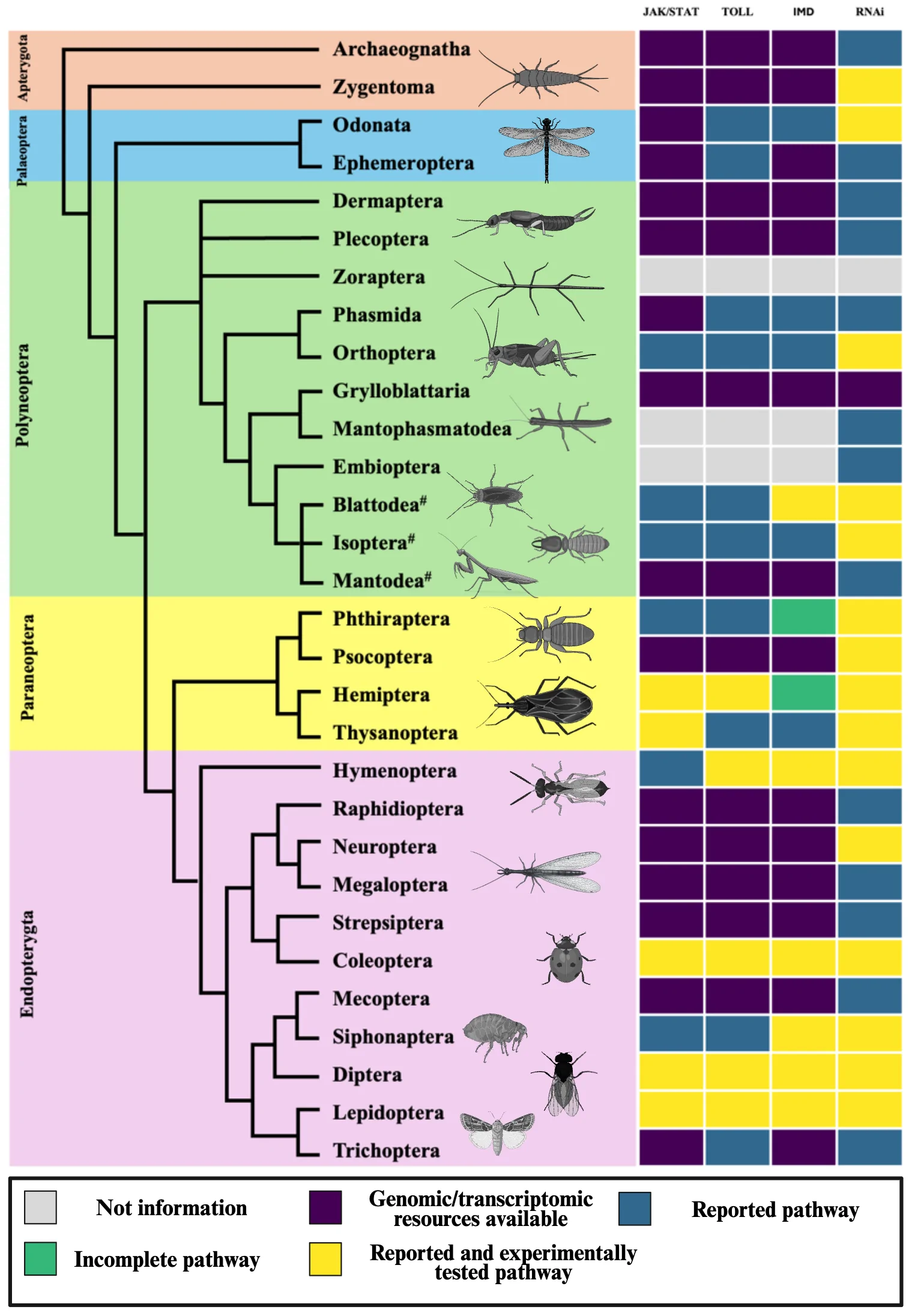
Comparative overview of the current knowledge of major innate immune pathways across insect orders. The image summarizes the reported presence, experimental validation, and available genomic or transcriptomic evidence for the JAK/STAT, Toll, IMD, and RNA interference (RNAi) pathways across insect orders. Colored boxes indicate the current level of evidence for each pathway within a given order: purple indicates genomic/transcriptomic resources available (raw unanalyzed data set), blue indicates a reported pathway (predicted genes from genomic/transcriptomic analysis, and the detection of transcript or protein), yellow indicates experimentally validated pathways (validated by functional assays such as gene silencing, genomic edition, and phenotypic changes, such as the RNAi pathways, in which its function could be validated but the pathways have not been necessary described), green indicates incomplete pathways (Most of the components are conserved, however, some key components from the pathway are missed), and gray indicates no available information (Not information at all). The phylogenetic relationships among insect orders are shown on the left is adapted from (50). The figure highlights the uneven distribution of immune pathway characterization across insects, with substantial functional information available for selected model orders such as Diptera, Lepidoptera, Coleoptera, and Hemiptera, while many understudied lineages remain poorly characterized. Created in BioRender. Maya-maldonado, K. (2026) https://BioRender.com/fqywi8y.

Similar to Diptera, the JAK/STAT pathway has been described and functionally characterized in several Lepidopteran species, including *Bombyx mori*, *Manduca sexta*, *Chilo suppressalis*, *Spodoptera frugiperda*, and *Plutella xylostella*. In these species, the core components of the pathway are typically represented by single-copy genes. However, Upd ligands remain poorly characterized and require further investigation. The JAK/STAT pathway in Lepidoptera plays a critical role in regulating immune responses against bacterial, viral, and fungal infections (33, 41–49) (**Figure 2**).

Hemiptera is the largest order within the Paraneoptera subclass and includes insects of major importance for agriculture and human health, such as aphids and triatomines. Most species within this order possess the core components of the JAK/STAT pathway; however, Upd ligands appear to be absent in all studied species (51–62). In some kissing bugs (triatomine species), key components are missing, including Hop in *Triatoma infestans*, STAT in *T. pallidipennis*, and Dome in *T. pallidipennis*, *T. dimidiata*, *T. infestans*, and *Rhodnius prolixus*. Additionally, the planthopper *Laodelphax striatellus* lacks a Hop homolog (59). Nevertheless, despite the absence of certain components, the pathway has been shown to respond to immune challenges and infections. Unlike the previous examples, some hemipterans exhibit gene expansion in the pathway. For example, the pea aphid *Acyrthosiphon pisum* possesses four copies of Dome and two STAT paralogues (54, 63), while the peach aphid *Myzus persicae* has two Dome and two STAT paralogues (57). The JAK/STAT pathway in Hemiptera has been described as essential for controlling viral, bacterial, and parasitic infections (49, 55, 58, 59). Interestingly, activation of the pathway by viral proteins through interactions with Dome has been reported (55), suggesting an adaptation in which a receptor typically functioning as a cytokine receptor can directly recognize viral components. The presence of multiple paralogues, the absence of some core components, and the non-canonical functions of the Dome receptor collectively suggest that hemipteran insects have evolved robust adaptive mechanisms to modulate one of the most conserved signaling pathways in animals (**Figure 2**).

Other orders within the Paraneoptera subclass include Thysanoptera (thrips), Phthiraptera (sucking and biting lice), and Psocoptera (booklice and barklice). Both Phthiraptera and Psocoptera include several lice species, such as the human lice *Pediculus humanus humans* and *Pediculus humanus capitis*, as well as the chicken louse *Menopon gallinae* and the booklouse *Liposcelis brunnea*. In all of these species, the JAK/STAT pathway has been described; they exhibit single-copy genes, except in *Liposcelis brunnea*, which has four copies of Dome. However, the pathway is yet to be experimentally validated in these insect orders (64, 65) Similarly, in thrips (order Thysanoptera), the JAK/STAT pathway is complete. *Frankliniella fusca*, *Frankliniella tritici*, *Frankliniella occidentalis*, and *Frankliniella intonsa* each possess a single copy of the core pathway genes (66–68). Additionally, in *F. occidentalis* and *F. intonsa*, a UPD gene has been reported, and its role in controlling viral infections has been experimentally validated in both species (**Figure 2**).

Hymenopteran insects include social species such as bees, ants, and wasps. While these insects have been widely used as models for the study of eusocial behavior (69), research on immunity has been largely focused on bees. In several bee species, including *Apis mellifera*, *Bombus terrestris*, *Apis florea*, and *Megachile rotundata*, reductions in several immune gene families have been reported (70). However, the JAK/STAT pathway remains highly conserved in most of these species, as well as the parasitoid wasp *Pteromalus puparum*, which typically has a single copy of each core component (69–74). In *A. mellifera*, a recent next-generation sequencing study reported the expansion of four *hop* genes (75), suggesting that similar expansions may occur in other bee species; further studies are required to validate this. While upregulation of the JAK/STAT pathway has been reported during viral infections, no functional studies demonstrating its role in the antiviral immune response have been performed (**Figure 2**).

Coleoptera comprises all beetles, and within this order, the JAK/STAT pathway is highly conserved, with all studied species harboring a single copy of each core component (Dome, Hop, and STAT) (76–79). This pathway has also been implicated in the regulation of fungal infections (78).

The superorder Dictyoptera comprises the orders Blattodea, Isoptera, and Mantodea, which are represented by cockroaches, termites, and mantises, respectively. While the JAK/STAT pathway has been described in detail in cockroach, *Periplaneta americana* (Blattodea), including the presence of a UPD gene (80, 81), it has only been partially characterized in termite, *Odontotermes formosanus* (Isoptera) (82), and remains unexplored in the order Mantodea (**Figure 2**).

Fleas belong to the order Siphonaptera. In *Xenopsylla cheopis*, the JAK/STAT pathway exhibits an expansion of Dome genes (three copies), while only a single copy of Hop and STAT is present, and Upd is absent (83). Although transcriptomic changes in JAK/STAT pathway genes during bacterial and fungal infections have been reported in several Orthoptera (grasshoppers, crickets, and bush-crickets), the pathway remains only partially characterized and further studies are required to better understand it and validate its role in immunity (84–87) (**Figure 2**).

### 2.1 Beyond its role in immunity, the JAK/STAT pathway also plays important roles in regeneration

The JAK/STAT pathway has functions beyond immunity; it is also involved in tissue regeneration (87). For example, in Orthoptera (*Gryllus bimaculatus*), the pathway plays a key role in leg regeneration has been functionally tested, while its role in immune responses remains unexplored (86). In *Drosophila* (Diptera), this pathway plays a critical role in tissue regeneration, where hemocytes secrete Upds to activate the pathway in the intestinal epithelium, thereby promoting cell proliferation and midgut regeneration (88).On the other hand, in *P. americana* (Dictyoptera: Blattodea), silencing of the Upd gene disrupts leg regeneration, suggesting that this pathway is involved in the process (82). Similarly, in lepidopteran insects such as *Chilo suppressalis* and *Spodoptera frugiperda*, the JAK/STAT pathway plays a critical role in intestinal epithelial regeneration (46).

Overall genomics and functional validation across Diptera, Lepidoptera, Hemiptera, Hymenoptera, Coleoptera, and other insect groups demonstrate its central role in immunity against viruses, bacteria, fungi, and parasitoids. Divergence in the JAK/STAT pathway is largely observed in the ligands (UPDs) that activate it (**Figure 1**). There appears to be a biological constraint on the core signaling pathway, as the complete absence of the pathway has not been reported in any insect order. This could be due to its role in development, regeneration, and tissue homeostasis. The only variation in the core pathway appears to be present as gene expansions and duplications in certain insect orders. The apparent absence of certain core pathway components in some Hemipteran insects suggests alternative regulatory mechanisms, as the pathway itself is still responsive. Based on current knowledge, the JAK/STAT pathway is among the most conserved signaling pathways in insects, and its functions are likely to be conserved across understudied orders and species.

## 3 Toll pathway

The Toll pathway was first established in fruit flies, *Drosophila melanogaster*, as a developmental pathway controlling dorsal-ventral embryonic polarity (89, 90). Its immune function was described later, when the spätzle/Toll/cactus cassette was shown to control the antifungal response in adult flies (91).

Components of the canonical Toll pathway include Spätzle, Toll, MyD88, Pelle, Cactus, and Dif/Dorsal. Several studies across insects have shown that the Toll pathway is broadly conserved and functionally active beyond Diptera. Functional validation has been reported across multiple orders, including Hemiptera, Hymenoptera, Coleoptera, and Lepidoptera, in which Toll signaling contributes to antibacterial, antifungal, antiviral, and antiparasitic immune responses. During an infection, the Toll pathway is activated by the recognition of pathogen-associated molecular patterns (PAMPs) by several pattern recognition receptors (PRRs). These receptors activate proteases such as ModSP and persephone, which cleave pro-Spz into its active form, Spz, which then binds to the Toll receptors. The activated intracellular domain of Toll receptors recruits MyD88; the adaptor protein Tube then binds to MyD88, recruiting the kinase Pelle, which phosphorylates the repressor Cactus, triggering its degradation and releasing Dif/Dorsal. Released Dorsal translocates to the nucleus and works as a transcription factor of immune genes (92, 93) (**Figure 1**).

The Toll pathway in Diptera appears broadly conserved, but not identical. The best conserved core is the intracellular components, including MyD88, Tube, Pelle, and Pellino, which were recovered across the dipteran genomes sampled in a broad comparative study (94), including mosquitoes, sand flies, tsetse, house flies, stable flies, blow flies, fruit flies, and other Drosophilids. The component that shows the greatest variation is the Toll receptor, with substantial differences in copy number between dipteran species, indicating that, in Diptera, despite the pathway being conserved at the signaling core and in immune function, the upstream receptor architecture has been remodeled repeatedly during dipteran evolution (**Figure 2**).

Several studies across Diptera have functionally demonstrated that the Toll pathway plays a central role in diverse immune responses. For example, in the mosquito *Aedes aegypti*, Toll signaling is functionally active against the dengue virus (95, 96). Moreover, in *Anopheles gambiae*, studies established that Toll signaling contributes to limiting parasite development during malaria infection (97). Beyond mosquitoes, comparative genomics of tsetse flies shows retained Toll and IMD immune gene families, supporting conservation of the pathway (98). Studies in sand flies provide functional evidence of Toll immunity. Interestingly, *Leishmania* suppresses Toll immunity in *Lutzomyia longipalpis* by modulating Toll pathway repressors, such as cactus and SHP-2, suggesting a conserved immunosuppression mechanism analogous to that observed in mammals (99, 100).

Available genomes, transcriptomes, and functional studies on Hemiptera consistently support the presence of a broadly conserved Toll signaling module, though with species-specific variation. In aphids such as *Acyrthosiphon pisum*, a near-canonical pathway has been described, including the main core components Spätzle, Toll, Tube, MyD88, Pelle, Cactus, and Dorsal, along with additional regulators such as Pellino and Traf, indicating that the full intracellular cascade is retained (63). Similar complements of core components have been reported in psyllids, *Diaphorina citri* (101), and triatomines, although some differences exist, including the apparent absence of Dif in *Rhodnius prolixus* (102). In addition, key components of the Toll pathway are differentially expressed in *R. prolixus* when the gut is infected with *Trypanosoma*, indicating that the pathway is functional during parasitic infections (59). In other insects from the order, such as planthoppers, including *Laodelphax striatellus* and *Sogatella furcifera*, main core Toll components, such as Toll receptors, MyD88, Tube, and Dorsal, have been directly implicated in antiviral responses, while in aphids, *Myzus persicae*, multiple Toll receptors are transcriptionally induced upon bacterial infection and are functionally relevant for host survival (101, 103, 104). However, in Coccidae, there is a partial reduction, with missing core elements such as MyD88 and Cactus, suggesting that although the pathway is retained, it may be modified in specific ecological contexts (55).

Overall, these data indicate that the Toll pathway in Hemiptera is conserved but evolutionarily flexible, maintaining a stable intracellular core while allowing diversification at the receptor and regulatory levels. This pattern contrasts with the frequent loss of the IMD pathway in the same order (discussed later) and suggests that Toll signaling fulfills essential roles that constrain its elimination. In fact, in Triatoma pallidipennis, cross-activation of Toll and IMD pathways has been reported in response to systemic infections (105). At the same time, variation in gene copy number, differential presence of specific components, and experimentally validated roles in antiviral and antibacterial defense indicate that the Toll pathway has been adaptively remodeled in response to species-specific ecological pressures, including symbiosis, parasitism, and host–pathogen interactions (**Figure 2**).

In other orders, such as Hymenoptera, Coleoptera, and Lepidoptera, the pathway’s functionality has been experimentally validated. In Hymenoptera, comparative genomic evidence supports a broadly conserved Toll signaling repertoire, including core intracellular components such as MyD88, Tube, Pelle, and Pellino, together with multiple Spätzle ligands and Toll receptors, although Toll9 appears absent in some hymenopterans (70, 94). In addition, functional studies in parasitoid wasps indicate that Toll signaling contributes to antifungal immunity (106). In Coleoptera, the Toll pathway is strongly supported by both comparative and functional evidence. Genomic analyses in *Onthophagus taurus* and *Tribolium castaneum* indicate the retention of core intracellular Toll components, along with multiple Toll receptors and Spätzle ligands (94). Moreover, studies in *T. castaneum* showed that antimicrobial peptide induction depends on the Toll and IMD pathways, supporting an active immune role for Toll signaling (107, 108). Similarly, an RNAi study showed that Toll signaling contributes to the induction of antimicrobial peptides and to defense against microbial infection (108). In Lepidoptera, the Toll pathway is strongly supported by both comparative and functional evidence. Genomic analyses indicate retention of the canonical intracellular signaling core together with multiple Toll receptors and Spätzle ligands (94), while experimental studies in *Bombyx mori* and *Manduca sexta* have demonstrated functional Toll-Spätzle signaling linked to antimicrobial peptide induction (48, 109). Additional work in *Spodoptera exigua* and *Plutella xylostella* further indicates that Toll signaling contributes to both humoral and cellular immune responses, supporting the view that this pathway is broadly conserved and immunologically active across Lepidoptera (110–112).

Despite overall conservation, comparative analyses reveal specific variations, particularly in the number and diversification of Toll receptors and Spätzle ligands, as well as occasional modifications in pathway components. Together, these works indicate that the Toll pathway represents a conserved but evolutionarily flexible immune signaling system across insects.

Several orders, including Orthoptera, Odonata, Phasmida, Blattodea, Isoptera, Ephemeroptera, Phthiraptera, Thysanoptera, Trichoptera, and Siphonaptera, possess genomic or transcriptomic evidence supporting the presence of a conserved Toll pathway. However, functional validation remains limited or lacking. In *Locusta migratoria* (Orthoptera) transcriptomic analyses identified Spätzle, Toll/TLRs, MyD88, Pelle, Pellino, Cactus, Dorsal/Dif, Tollip, TRAF2, and several of these genes changed expression after fungal infection (85). Additional studies in locusts and crickets also annotated canonical Toll components and measured gene expression (84, 113, 114). However, Tube was not detected in the main locust dataset, and there is still no clear pathway-specific functional validation comparable to the canonical Dipteran pathway. Similarly, in Odonata, genomic resources have revealed the presence of core signaling factors, including *dorsal*, *cactus*, *Traf6*, *pelle*, *Myd88*, and Toll-like receptors (94, 115, 116). In addition, available evidence in Phasmida supports the presence of a Toll-related immune signaling system. In particular, for *Peruphasma schultei*, microbial challenge induces a diverse antimicrobial peptide repertoire and several Toll-like receptors (117). while in *Bacillus rossius*, transcriptomic analyses identified Spätzle, Toll, MYD88, Pelle, and Cactus, together with additional Toll-like receptors. However, the apparent absence of Tube and the lack of pathway-specific functional assays indicate that Toll signaling in both orders is not fully characterized (118).

In Blattodea, the Toll pathway appears to be broadly conserved. Genomic and comparative studies in *Blattella germanica* and *Periplaneta americana* support an expanded immune gene repertoire and retention of key Toll signaling components, although detailed functional dissection of canonical Toll signaling remains comparatively limited (80, 119, 120). Similarly, in Isoptera, available comparative and expression-based studies support the presence of a conserved Toll pathway. As such, termite genomes retain canonical immune signaling genes, and studies in *Reticulitermes chinensis* have reported Toll-related components, including *Toll9, TRAF6, MyD88*, and *Pellino* (94, 121, 122). Notably, in Ephemeroptera, the genome of *Ephemera danica* supports a diversified Toll signaling repertoire, including MyD88, Tube, Pelle, Pellino, multiple Spätzle genes, and 22 Toll receptors, with a notable expansion of the Toll9 subfamily (94) (**Figure 2**).

Some studies suggest that Toll-related immunity is conserved in Phthiraptera; however, it has been poorly characterized. For example, the genome of *Pediculus humanus* contains coding sequences of the canonical intracellular Toll components (MyD88, Tube, Pelle, Pellino), together with 8 Spätzle and 6 Toll genes (94). Moreover, infection with *Bartonella quintana* upregulates *Spaetzle* and *Defensin 1* expression, consistent with Toll pathway activation (123). Thus, preliminary evidence supports that immunity-related gene families remain conserved across parasitic lice. In contrast, orders such as Phthiraptera, Thysanoptera, and Siphonaptera exhibit evidence of Toll retention accompanied by specific remodeling. Similarly, in (17) *Frankliniella occidentalis*, duplicated intracellular components (*MyD88, Tube, Pelle*) and expanded ligand repertoires indicate diversification, with transcriptomic studies linking Toll components to antiviral responses (94, 124). In fleas such as *Ctenocephalides felis*, the core intracellular module is conserved but with modifications in Toll receptor repertoire, and current functional studies suggest that immune responses may rely mainly on alternative pathways such as IMD. Finally, in Trichoptera, computational evidence from *Limnephilus lunatus* indicates partial conservation of Toll signaling, although missing components and lack of functional validation highlight the need for further investigation (94).

On the other hand, understudied orders such as Archaeognatha, Zygentoma, Dermaptera, Plecoptera, Grylloblattodea, Psocoptera, Raphidioptera, Neuroptera, Megaloptera, Strepsiptera, and Mecoptera have available omics resources; nevertheless, there are currently no studies specifically addressing the annotation or functional characterization of the Toll pathway. In Mantodea, the presence of Toll signaling is plausible based on comparative analyses and the recent availability of high-quality genomes, but direct evidence remains scarce. Overall, these cases highlight that while genomic resources are increasingly available, functional studies are needed to fully characterize the Toll pathway across these understudied orders (**Figure 2**).

Taken together, these observations reveal a consistent pattern in which the Toll pathway is deeply conserved across insect evolution, even in highly divergent orders having different ecology. The recurrent detection of core intracellular components, including *MyD88, Tube, Pelle, Pellino*, and *Dif/Dorsal*, suggests a strong evolutionary constraint on the signaling backbone of the pathway. Similar to the JAK/STAT pathway, the observed variation seems to be upstream of the pathway where Toll receptors and Spätzle ligands show more divergence and flexibility. Currently limited evidence from functional assays in understudied orders highlights a major gap in our understanding. Future studies need to integrate functional genomics, RNAi, and infection assays across these understudied orders to determine if the Toll pathway operates through conserved mechanisms or has undergone lineage-specific variations in response to ecological pressures such as parasitism, symbiosis, or habitat specialization.

## 4 IMD pathway

The immune deficiency (IMD) pathway regulates the activity of the transcription factor Relish, which controls the expression of most of the AMPs, including cecropin, diptericin, drosocin, and defensin (125). Activation of IMD involves pattern recognition receptors that can detect trace amounts of diaminopimelic acid (DAP)-type peptidoglycan, an essential component of the peptidoglycan of most Gram-negative bacteria and certain Gram-positive bacteria (126). In *Drosophila*, activation of the IMD pathway in the fat body leads to systemic AMP expression, although the pathway can also be activated locally in multiple tissues. Importantly, IMD activation in *Drosophila* is tissue-specific, with only a limited set of universal IMD pathway targets, mainly AMPs, being shared across tissues, while the response leads to the expression of infection-induced genes that exhibit tissue-restricted expression patterns (127).

This pathway resembles the mammalian Tumor Necrosis Factor Receptor (TNFR) signaling pathway, which is involved in inflammation (126). In insects, the IMD pathway is highly conserved in structure and function, triggering the production of AMPS, which highlights its functional conservation in innate immunity. Although the core elements are shared, they are not identical across insect orders and species. For example, based on the *Drosophila* IMD pathway, core proteins include PRRs, such as PGRPs, the adaptor protein initiating intracellular signaling IMD, the caspase-8 homolog FADD, the Death-related ced-3/Nedd-2-like protein DREDD, the transforming growth factor-activated Kinase 1 TAK1, the inhibitor of κB kinase IKK complex, and the NF-κB family transcription factor Relish. Most of them have homologs across a wide range of insect taxa. The pathway is activated after (DAP)-type peptidoglycan binds to PGRPs, which activate the IMD, FADD, and DREDD cascades; activated DREDD cleaves IMD, and cleaved IMD recruits TAK1, which activates the IKK complex. Finally, Relish is activated by phosphorylation of IKK and cleavage by DREDD, leading to its nuclear translocation and regulation of immune gene expression [see detailed in (126)] (**Figure 1**).

### 4.1 Canonical IMD pathway across insects

Orders Diptera, Coleoptera, Hymenoptera, Lepidoptera, and Siphonaptera show strong evidence for a conserved and functional IMD pathway. In *Drosophila melanogaster*, the pathway is well-established as a major regulator of antibacterial immunity (125), while studies in mosquitoes such as *Anopheles gambiae and Aedes aegypti* further demonstrate conserved roles in antimicrobial defense, anti-parasite responses, microbiome homeostasis, and anti-fungal responses (128–130). Similarly, in Coleoptera, genomic and experimental studies in beetle models such as *Tribolium castaneum* and *Tenebrio molitor* demonstrate that key core components of the IMD pathway regulate antimicrobial peptide expression and contribute to antibacterial immunity (79, 107, 131–136).

Likewise, in *Apis mellifera* (Hymenoptera), genomic and infection-based studies support the presence and inducibility of core IMD components, including *PGRP-LC, imd, Tak1, Dredd, Kenny*, and *Tab*, supporting roles in antimicrobial defense (74, 137). Besides bees, the presence of the IMD pathway is further supported in other hymenopterans. As such, in *Bombus terrestris*, infection induces *relish* and AMP expression in a nutrient-dependent manner (72). Interestingly, in eusocial Hymenoptera, such as ants, *Solenopsis invicta*, viral infection reshapes innate immune gene expression and implicates IMD-related signaling (138). Moreover, in the ant *Lasius niger*, recent genomic and functional analyses indicate that the IMD pathway is structurally complete and contributes to individual antibacterial immunity (139). Together, these studies support that the hymenopteran IMD pathway is conserved but ecologically modulated across lineages (**Figure 2**).

In Lepidoptera, genomic, transcriptomic, and functional studies in species such as *Bombyx mori*, *Spodoptera exigua*, and *Manduca sexta* support the presence of core IMD components and their roles in antimicrobial peptide regulation, although aspects of pathway activation and downstream antimicrobial peptide induction appear to be different from the canonical dipteran model due to extensive crosstalk with other immune pathways (140–144). In addition, for Sipheonptera, the IMD pathway is present and functional, at least in the cat flea *Ctenocephalides felis*, where homologs of the core IMD components have been identified, and knockdown of *imd* or *Relish* increases bacteria burden (145). Moreover, transcriptional induction of the IMD signaling pathway in response to bacterial exposure in the flea gut provides additional evidence that the IMD response is active in flea gut immunity (146). Despite broader comparative data across fleas remaining limited, available evidence supports the presence of a functional IMD pathway in other flea species (83) (**Figure 2**).

### 4.2 Partial IMD pathway conservation in some insect orders; do symbionts play a role?

Orders Hemiptera and Phthiraptera show evidence for a partial or reduced IMD Pathway. For Hemiptera, the IMD pathway appears to be absent or incomplete in several lineages, with canonical components missing in some taxa. For example, IMD-related genes in aphids include PGRPs, IMD, dFADD, Dredd, and Relish (63). Aphids have a primary symbiotic association with bacteria from the genus *Buchnera*. Other bacteria, like Hamiltonella, Serratia also serve as secondary symbionts in aphids (147, 148). Aphids use plant sap as their primary food source, which is nutrient poor and sterile, limiting microbial exposure. Due the lack of essential nutrients in food, aphids depend on their symbionts to provide nutrients required for survival and reproduction (149). It is hypothesized that this symbiotic association is primarily responsible for the loss of IMD pathway related immune functions (150). More recently, another work found that the same core elements of the IMD pathway are absent on soft scale insects (Hemiptera: Coccidae) (55). Weather the reduction in their IMD pathway functionality observed in scale insects is due to symbiotic association bacteria remains unclear. Other hemipterans, such as the Asian citrus psyllid, *Diaphorina citri*, also lack a portion of the IMD pathway (53). Similar to aphids and scale insects, the loss of IMD pathway is also attributed to symbionts. In triatomines, initial genome analyses questioned the existence of a functional IMD pathway because several core components, including IMD, Fadd, and Dredd, were absent. However, the presence of PGRPs and a *Relish* homolog suggested that IMD signaling might still operate through an alternative noncanonical mechanism (151). Later bioinformatic analyses identified nearly all previously missing core IMD pathway components in *Rhodnius prolixus*, except IMD and Kenny, indicating that alternative functional activation of IMD exists in *R. prolixus* (152, 153). Despite the absence of conventional IMD pathway, some hemipteran insects harbor functionally conserved but sequentially diverse IMD genes. For example, brown-winged green stinkbug *Plautia stali*, exabits a fully functional IMD pathway with low similarity to known IMD genes (154). These non-canonical genes are also present in bedbug and brown marmorated stinkbug (155). This study showed that the innate immune system, particularly the IMD pathway, is highly diverse in hemipteran insects. It highlights that previously overlooked IMD pathway genes in bedbugs and stinkbugs that had been missed because their DNA and protein sequences were highly divergent from known IMD genes. This suggests that some hemipteran groups considered to lack the IMD pathway may possess highly divergent novel versions of these genes (**Figure 2**).

Similarly, Phthiraptera exhibits a reduced immune gene repertoire, and available lice genomes support loss of key IMD components, including IMD and Fadd, indicating an incomplete IMD pathway (64, 65). The same occurs in Thysanoptera, where available genomic and transcriptomic resources indicate that the canonical IMD pathway may be incomplete or divergent. In particular, for *Frankliniella occidentalis*, neither IMD nor FADD has been detected, although other immunity-related genes and IMD-associated transcriptional responses have been reported (156).

### 4.3 Conserved IMD signaling with limited functional validation

In the orders Odonata, Orthoptera, and Phasmida, several core genes of the IMD pathway have been identified but not functionally tested to date. As such, key components of the Imd pathway are present in Odonata species (124, 125). Similarly, pattern-recognition receptors and downstream immune genes related to the IMD pathway have been identified in Orthopteran species through transcriptomic analyses, supporting conservation of antibacterial signaling pathways in this order (157, 158). In the order Phasmida, a broad spectrum of antimicrobial peptides and pattern-recognition proteins, including peptidoglycan-recognition-related proteins, was identified following immune challenge in *Peruphasma schultei* (109). Although transcriptional profiles provide strong support for the existence of core immune genes in the IMD pathway, they have not yet been fully mapped.

The order Dictyoptera is collectively formed by Blattodea (cockroaches), Isoptera (termites), and Mantodea (mantises). Nearly all canonical intracellular and upstream receptor modules of the IMD signaling cascade were detected and expressed across multiple tissues in the adult German cockroach, *Blattella germanica*, except for IKK-related genes, indicating functional activation of the IMD pathway in cockroaches (159). Similarly, genomic analyses strongly suggest the presence of homologs based on comparative annotation and thus the retention of immunity pathways, including IMD, in termites (119, 160, 161). Although comparisons of immune gene repertoires between termites and cockroaches indicate reduced diversity of immune genes. For example, PGRP genes are notably reduced in number in many termite species (119). Such differences might be due to lifestyle differences. For example, cockroaches are exposed to high pathogen loads and harbor a highly complex gut microbial community, including Gram-negative bacteria that are key activators of the IMD pathway, which may strongly favor the retention of IMD signaling genes. On the other hand, termites show reduced immune gene diversity associated with sociality and colony-level defense (119).

Given the close evolutionary relationship within the order Dictyoptera, Mantids might retain the IMD pathway. However, as in other orders, for Mantodea, although a genome exists, IMD genes have not been annotated or identified, limiting our knowledge in this suborder (162). Importantly, unlike termites, mantids are solitary predatory insects with relatively controlled diets and are thus not exposed to high microbial loads. Considering that natural selection often removes unnecessary genes, if confirmed, a reduction in IMD pathway-related genes in Mantodea may be expected and reflect ecological specialization associated with their lifestyle.

From an evolutionary point of view, the IMD pathway appears to be an ancient and conserved immune mechanism that has changed in different ways across insect groups. In some insect orders, certain genes have been lost, duplicated, or modified, while in others, the pathway may be activated through noncanonical mechanisms or interact more strongly with other immune pathways. These differences could be related to the particular ecology of each group, including their exposure to microorganisms, diet, microbiome composition, lifestyle, and degree of social organization.

## 5 RNA interference pathways in insects

RNA interference (RNAi) was first identified as a sequence-specific gene silencing mechanism, but it is now widely recognized as one of the primary antiviral defense systems in insects. Early studies showed that double-stranded RNA (dsRNA) can trigger targeted degradation of RNA molecules, meaning the cell can recognize and break down specific genetic material based on sequence matching (163, 164). This mechanism was later linked to antiviral immunity, as viral replication produces dsRNA intermediates that activate RNAi responses, allowing insects to detect and limit viral infections before they can spread.

In addition to its role in antiviral immunity, the RNAi pathway also helps regulate transposable elements and controls gene expression at the post-transcriptional level. The RNAi machinery is further classified into three major types: the small interfering RNA (siRNA) pathway, the microRNA (miRNA) pathway, and the PIWI-interacting RNA (piRNA) pathway. The siRNA pathway serves as the primary antiviral defense across all insect orders. The miRNA pathway indirectly contributes to antiviral defense by regulating gene expression during viral infections.

### 5.1 The siRNA pathway: core antiviral mechanism

**Figure 3:**
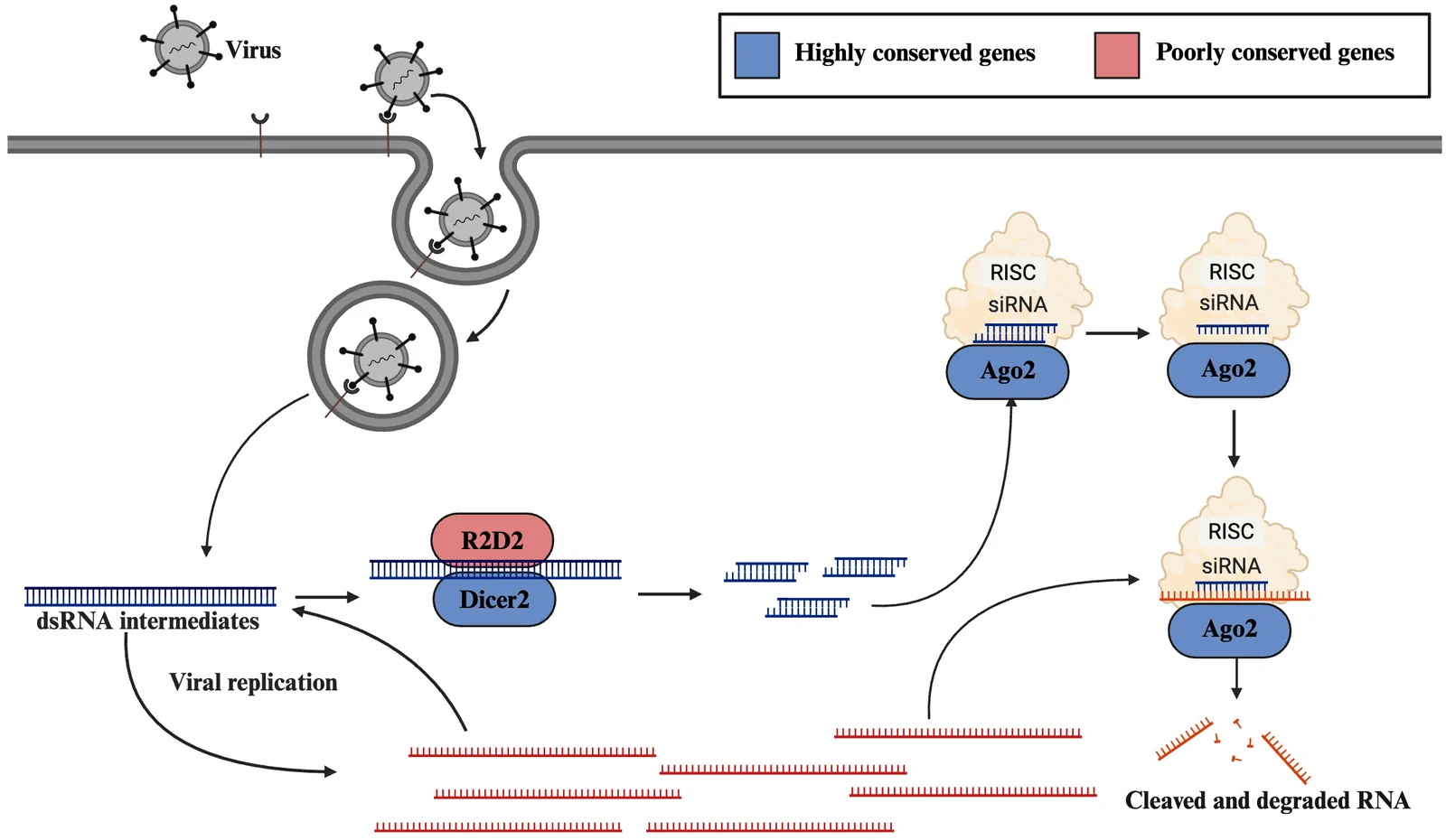
Overview of the small interfering RNA (siRNA) pathway in insects. The schematic illustrates the major components and antiviral functions of the insect siRNA pathway. The image represents a generalized model based on currently available evidence across insects. Created in BioRender. Maya-maldonado, K. (2026) https://BioRender.com/81gyrej.

In insects, the antiviral RNAi response is mainly driven by the small interfering RNA (siRNA) pathway. This pathway begins when dsRNA is recognized and cleaved by Dicer-2 (Dcr2), producing short interfering RNAs (siRNAs), which are small RNA fragments that guide silencing. These siRNAs are then loaded into the RNA-induced silencing complex (RISC), where Argonaute-2 (Ago2) uses sequence matching to identify and cleave viral RNA, effectively preventing viral replication (164–166). Together, Dcr2 and Ago2 make up the core of the antiviral RNAi response (**Figure 3**). Dcr2 also functions as a viral sensor in some insects and can activate additional antiviral responses beyond RNA cleavage. For example, in mosquitoes, Dcr2mediated recognition of viral dsRNA can stimulate the production of additional antiviral molecules, indicating that Dcr2 functions not only in RNA cleavage but also contributes to the early recognition of viral infection (30). These observations suggest that RNAi does not act independently but instead works together with other immune pathways to limit viral replication.

### 5.2 Role of R2D2 in antiviral RNAi

The dsRNA-binding protein R2D2 plays an important role in this pathway by stabilizing siRNAs and loading them onto Ago2, which is required for RNA cleavage. Because of this role, Dcr2, Ago2, and R2D2 are commonly presented as the main functional components of antiviral RNAi (30). However, this model does not fully reflect how the pathway works across insect diversity. Comparative studies show that R2D2 is not present in all insect lineages and likely originated later in evolution, specifically within winged insects (Pterygota) (167). This means that some insects still carry out RNAi even when R2D2 is reduced or absent, suggesting that other proteins, such as Loquacious, help maintain the pathway in those species.

#### 5.2.1 Functional role of loquacious

Loquacious (Loqs) is another dsRNA-binding protein that works closely with Dicer and Argonaute proteins and is involved in multiple RNA processing pathways. Instead of functioning as a single-purpose protein, Loqs can bind RNA and help guide it through different processing steps, suggesting that the RNAi pathway is not strictly dependent on R2D2 for function. In species where R2D2 is reduced or absent, Loqs may help support similar processes in the pathway, such as stabilizing RNA molecules or facilitating the transfer of Argonaute proteins for silencing (167). This shows that insects can maintain the overall RNAi function by using different proteins that perform the same roles. This allows the pathway to remain conserved even when certain components differ or are lost across lineages. Together, these findings suggest that the RNAi pathway is not dependent on one fixed set of proteins. Different insect groups appear to use slightly different proteins to accomplish the same overall task. This may help explain how RNAi has remained functional throughout insect evolution, even though some proteins have been gained, lost, and modified over time. Rather than being identical in every species, the pathway appears to have changed in different lineages while preserving its role in antiviral defense.

#### 5.2.2 The piRNA pathway

In addition to the siRNA response, insects also possess the PIWI-interacting RNA (piRNA) pathway. This pathway primarily controls transposable elements, which are mobile genetic elements that can disrupt normal gene function. Unlike the siRNA pathway, the piRNA pathway does not rely on Dicer enzymes. Instead, it uses PIWI proteins and a self-amplifying process, often referred to as the “ping-pong” cycle, where RNA molecules are repeatedly processed to strengthen the silencing signal (168). Studies in mosquitoes have suggested a possible antiviral role of piRNA pathway in antiviral defense. Suggesting that insects may use multiple RNA interference mechanisms simultaneously to control infection (156, 169). Studies in mosquitos have shown that the piRNA pathway can also contribute to antiviral defense, suggesting that insects may rely on multiple RNA interference mechanisms simultaneously to control infection (169, 170).

### 5.3 RNAi pathways across insect orders

Although the core RNAi machinery is conserved, its effectiveness varies across insect groups. These differences are usually not due to the absence of the pathway itself, but rather differences in how dsRNA enters cells, how long it remains stable, and how efficiently it is processed and spread throughout the organism (**Figure 2**).

In Diptera, RNAi is one of the best-characterized antiviral defense systems. In mosquitoes such as Aedes aegypti and Anopheles gambiae, the siRNA pathway plays an important role in controlling arbovirus infections. The core components Dcr2 and Ago2 are conserved and work together to suppress viral replication by recognizing and degrading viral RNA (30, 166). Studies show that when Dcr2 is disrupted, viral replication increases, especially during the early stages of infection, which shows its role in limiting how quickly a virus can begin replicating within the host (171).

R2D2 also contributes to this process, but its role extends beyond RNAi alone. In *Aedes aegypti*, partial loss of R2D2 leads to increased viral replication, whereas complete loss appears lethal, meaning the organism cannot survive without it (172). This suggests that R2D2 is not only involved in antiviral defense but also in essential biological processes, such as development and reproduction. Also, proteins like Loqucious can interact with viral RNA proteins, thereby influencing how viruses replicate within the cell, not just how RNA is silenced (173). This shows that proteins involved in RNAi do not have a single specific role. In addition to helping processes and silence viral RNA, proteins like R2D2 and Loquacious can also influence processes such as development, reproduction, or replication itself, depending on the species.

One important limitation in Diptera is the reduced ability for RNAi signals to spread between cells. This is partly due to the loss of SID-1 proteins, which normally facilitate the transport of dsRNA across cell membranes, allowing multiple cells to activate the RNAi response. Without this transport system, RNAi responses tend to remain localized to the cells where dsRNA is first introduced (167, 174). As a result, RNAi in these insects is mostly limited to the cells that first take up dsRNA, so neighboring cells and tissues do not receive sufficient signal to activate gene silencing. Interestingly, the loss of SID-1 does not necessarily mean the systemic RNAi is absent. Studies in locus have shown that RNAi signals can spread throughout the body even without SID-1 homologs, suggesting that other mechanisms are involved in transporting dsRNA between cells (122). These findings indicate that insects may have evolved multiple ways to achieve systemic RNAi and that the movement of RNAi signals is likely more complicated than originally thought.

In contrast to Diptera, Hemiptera tend to exhibit a different pattern, in which RNAi signals can move more effectively between cells. Studies on species such as the soybean aphid (*Aphis glycines*) have identified core RNAi components, including Dcr2 and Ago2, as well as SID-1 genes, which facilitate the movement of dsRNA between cells and spread the RNAi signal more effectively (175). Furthermore, SID-1 functions as a channel that allows long dsRNA molecules to cross cell membranes (176). However, RNAi efficiency still varies within this group, indicating that even when dsRNA can move between cells, other factors, such as degradation, uptake, or processing, can limit the effectiveness of the RNAi pathway in silencing target RNA or controlling infection (174).

Compared with Diptera, many hemipteran insects have retained SID-1 genes, which may allow RNAi signals to spread more efficiently between cells. This suggests that different insect groups have evolved different strategies for transporting dsRNA. The presence of SID-1 alone does not guarantee strong RNAi responses, since factors such as dsRNA degradation and cellular uptake can also influence how effectively the pathway functions.

Coleopteran insects generally exhibit strong, consistent RNAi responses. In species such as Tribolium castaneum, dsRNA is efficiently taken up by cells, processed by Dcr2, and spread throughout the organism, resulting in reliable gene silencing (177). This shows that when dsRNA can enter cells, remain stable long enough to be processed, and spread between tissues, the RNAi pathway can consistently generate sufficient siRNAs to silence target genes across the organism (177). As a result, coleopterans are widely used as model systems for RNAi-based applications. Overall, coleopteran insects appear to have some of the strongest RNAi responses among insects. Efficient uptake of dsRNA, strong systemic responses, and reliable gene silencing have made beetles important for RNAi research. As a result, coleopterans are widely used as model systems as they are attractive targets for RNAi-based pest control strategies.

In Lepidoptera, RNAi responses are often weaker and less consistent, with gene silencing working in some species but failing or being much less effective in others. One of the main factors contributing to this reduced efficiency is the presence of dsRNases, enzymes that degrade dsRNA in the gut and hemolymph before it can be processed by Dcr2 (178). This means that even though the pathway is present, it is harder to activate because the dsRNA signal is lost before it can be used. In addition, reduced uptake and limited spread of dsRNA can also contribute to lower RNAi efficiency in this group (174). So, the RNAi pathway is present but functions poorly, largely due to rapid dsRNA degradation and reduced uptake and spread in Lepidoptera. Taken together, these studies suggest that reduced RNAi efficiency in Lepidoptera is not caused by the loss of the pathway itself. Instead, several factors appear to limit its effectiveness. Rapid degradation of dsRNA, reduced uptake, and limited movement between tissues all contribute to weaker and less predictable RNAi responses compared with those observed in coleopterans or hemipterans.

Most of what we currently know about RNAi comes from studies in Diptera, Coleoptera, Hemiptera, and Lepidoptera. In many other insect orders, including Hymenoptera, Orthoptera, Blattodea, and Isoptera, RNAi has mainly been examined through genome and transcriptome sequencing rather than experiments directly testing how the pathway functions during infection. Because of this, it is often possible to identify genes associated with the RNAi pathway without knowing whether they perform the same role in every species. In addition, incomplete genome assemblies, differences in gene sequences, and difficulty matching genes across distantly related insects can make some RNAi components appear to be absent when they have simply not been identified yet. As more insect genomes become available and additional functional studies are completed, our understanding of how RNAi operates across these insect orders will continue to improve.

Compared with Diptera, much less is known about RNAi in Orthoptera. Studies in locusts have shown that systemic RNAi can occur even in the absence of SID-1 homologs (179). These findings suggest that insects may have different mechanisms to transport dsRNA between cells. This also indicates that the ability to spread RNAi signals throughout the body has been conserved even though the proteins involved may differ among species. Additional studies will be needed to determine whether similar mechanisms occur in other orthopteran insects.

In Hymenoptera, including bees and parasitoid wasps, genes associated with the RNAi pathway have been identified, although relatively few functional studies have been done. Most of what is currently known comes from genomic and transciptomic studies rather than experiments directly testing antiviral responses. As a result, while these studies suggest that the core components of RNAi are present in hymenopteran insects, additional work will be needed to determine how these pathways function during viral infection and whether they perform similar functions to those described in other insect order.

### 5.4 Evolutionary patterns

These patterns show that RNAi is both conserved and adaptable across insect evolution. The core function of the pathway, recognizing dsRNA and silencing specific RNA sequences, is maintained across insect orders. However, the specific proteins and processes that support this function have changed over time. Studies comparing RNAi genes across insects have shown that the pathway has changed over time rather than remaining identical across lineages. For example, R2D2 appears to have emerged later in insect evolution, while SID-1 homologs have been lost in Diptera (167). Even with these differences, insects are still able to recognize and silence foreign RNA. This suggests that there is not one universal version of the RNAi pathway shared by all insects. Instead, different insect groups appear to use slightly different proteins and mechanisms to accomplish the same general function. Therefore, RNAi seems to have been shaped by the evolutionary history of each lineage, while maintaining its role as an important antiviral defense mechanism. However, much of this evolutionary framework is based on comparative genomic studies, and functional evidence is still limited outside of a relatively small number of model insects. As additional species are studied, our understanding of how RNAi has evolved across insects will likely continue to change.

#### 5.4.1 Viral suppressors of RNAi

Although RNAi provides an effective defense against viral infection, many viruses have evolved mechanisms to counter this pathway. Some viruses encode viral suppressors of RNAi (VSRs), which interfere with different stages of RNAi, including dsRNA recognition, Dcr2 processing, and Ago2-mediated cleavage (166). These proteins allow viruses to avoid degradation and continue replicating inside the host. The precision of VSRs suggests that RNAi places strong selective pressure on viruses, and in turn, viruses have evolved strategies to overcome this defense. This back-and-forth interaction is the evolutionary history between insect viruses and RNAi-mediated immunity (180).

### 5.8 Implications for RNAi-based pest control

Overall, the literature generally agrees that the RNAi pathway is present and functional in all insect orders, but its function differs across species, although detailed functional studies are still limited to a small number of model species. Even though insects differ in how they uptake dsRNA, process it, and spread the signal between tissues, the ability to recognize and silence foreign RNA appears to be preserved throughout insect evolution. At the same time, differences in pathway components among species suggest that RNAi has continued to change over time rather than remaining identical across insect lineages. strategies, since the success of these approaches depends on how effectively particular species can take up and process dsRNA to silence target genes (181, 182).

## 6 Concluding remarks: could omics datasets help fill knowledge gaps in immunity across some insect orders?

Although the JAK/STAT, Toll, IMD, and RNAi immune pathways have been completely described and functionally characterized in several insect orders, they remain poorly studied in many others. Although recent genomic resources are available for Archaeognatha Zygentoma, Plecoptera, Dermaptera, Mecoptera, Megaloptera, Neooiptera, Raphidioptera Trichoptera, and Psocoptera, core IMD pathway components have not yet been explicitly reported or functionally characterized (183–196).

Despite limited information, the available genomic and transcriptomic resources have provided some insights into the immune responses of these orders. For example, recent *in silico* screening of available transcriptomic resources for early-branching insect lineages considered primitive, including Archaeognatha and Zygentoma,has revealed the presence of diverse and lineage-specific antimicrobial peptides, indicating that functional innate immune effector systems were already established early in insect evolution (183). However, these studies have not yet resolved the underlying immune signaling, and canonical immune pathways remain uncharacterized in these lineages. In addition, the gut of *Lepisma saccharina (Zygentoma)* harbors symbiotic microbes that aid digestion (197). Although only hemocyte populations are reported as part of their immune response in these primitive insects, microbial interactions in the gut may drive an immune response via the IMD pathway (198).

Similarly, in mayflies, the presence of the Imd pathway is currently unknown. However, in *Cloeon dipterum*, a recent study identified a cluster of genes highly expressed during the nymphal (aquatic) stage that is enriched for defense-related GO terms (199), including response to fungus (GO:0009620, GO:0050832), defense response to Gram-positive bacterium (GO:0050830, GO:0050829), and defense response to bacterium (GO:0042742), suggesting that immune responses at nymphal stage may be active in Ephemeroptera.

In addition, a transcriptomic dataset across multiple Grylloblattodea species provides insights into the expression of immune-related genes (200). Similarly, in Strepsiptera, recent genomic resources are available (201), and a comparative AMP analysis suggests that some strepsipteran species may show a very reduced antimicrobial peptide repertoire, but these results remain insufficient to infer loss of the IMD pathway itself (202). Further analysis of genomic resources is needed to identify immune pathways, including IMD genes.

In contrast, for Embioptera, there is insufficient genomic or transcriptomic data. However, embiopterans host a diversity of parasitoids, including dipteran parasitoids of the family Tachinidae (203). In other parasitoid-host systems, a transcriptomic study on *Bombyx mori* parasitized by tachinids showed upregulation of antimicrobial peptides, strongly suggesting activation of IMD-associated genes (204). Parasitoid associations in Embioptera suggest that parasitoid pressure could represent an important selective force shaping immune responses (205). Thus, immune pathways may be diversified in this order, potentially driven by parasitoidmediated selection rather than microbial exposure alone. For Mantophasmatodea and Zoraptera,genomic or transcriptomic resources for analyzing immune pathway gene repertoires remain extremely limited. Thus, no general immune responses have been reported in the literature.

Overall, based on the currently available evidence, innate immune pathways are present in insects with variations broadly associated with specific insect orders and, in some cases even particular species. Interactions with symbionts, diet, habitat, and parasite pressure are primary drivers behind this divergence. Most of our knowledge is currently derived from studies on selected model insects, primarily from Diptera and Coleoptera. Immune pathways remain poorly characterized in many understudied insect lineages. Furthermore, the immune pathway act as an interconnected network, however, the current studies about the immune pathway crosstalk, and interaction between cell types and immune tissues is limited to just to some species mostly from Diptera order. Recent advances in genomics and transcriptomic datasets will likely unravel lineage-specific immune adaptations. As demonstrated by certain studies, an apparent absence of certain immune pathways might be due to annotation limitations because of sequence divergence, rather than an actual loss of the pathway.

The emerging approaches, such as Next Generation Sequencing systems, and Genome Editing Technologies, absolutely will accelerate our knowledge about the molecular mechanisms in all living organisms, including immune pathways of insects. However, is important to highlight that we currently have transcriptomic and genomic data sets of 27 from 30 insect orders, many of which have not been analyzed (See at **Supplementary Table 1**), therefore doing so would help to fill many gaps across all insect orders in the short term. For future studies, it will be paramount to combine genomic, transcriptomic, and functional validation studies to truly understand immune system divergence in insects. A comparative analysis about immune pathway across the insect orders will help to identify essential components for all the insect or specific genes keys for the immune response of single species. This knowledge would help to develop more specific strategies to for example, to control an vector or pest specie with a minimum or not risk affecting other insect species.
