# Supplementary material for: Insect immune systems: same same but different but still same
Source: Front Immunol. 2026 Aug 13;17:1884037. doi: 10.3389/fimmu.2026.1884037 (PMC13535093; doi:10.3389/fimmu.2026.1884037)
Supplement: Supplementary file 1 [file Table1.pdf]

Supplementary Table 1. Insect orders with available transcriptomic or genomic resources for which immune pathways have not yet been analyzed.

Insect order	Immune pathway no analyzed yet	Resource available	References
Archaeognatha	JAK/STAT, Toll, IMD	Transcriptome	Gerdol M., 2026
Zygentoma	JAK/STAT, Toll, IMD	Transcriptome	Gerdol M., 2026
Odonata	JAK/STAT	Transcriptome	Chauhan et al., 2016
		Genome	Ioannidis et al., 2017
Ephemeroptera	JAK/STAT, IMD	Genome and transcriptome	Almudi et al., 2020
Dermaptera	JAK/STAT, Toll, IMD	Transcriptome	Roulin et al., 2014
		Genome	Kobayashi et al., 2023
Plecoptera	JAK/STAT, Toll, IMD	Genome	Macadam et al., 2022
		Genome	Mo et al., 2025
Phasmida	JAK/STAT	Transcriptome	Wu et al., 2016
Grylloblattaria	JAK/STAT, Toll, IMD, RNAi	Transcriptome	Schoville et al., 2021
Mantodea	JAK/STAT, Toll, IMD	Genome	Goldberg et al., 2024
Psocoptera	JAK/STAT, Toll, IMD	Genome	Feng et al., 2022
Raphidioptera	JAK/STAT, Toll, IMD	Genome	Wolf et al., 2024
		Genome	Shen et al., 2024
Neuroptera	JAK/STAT, Toll, IMD	Genome	Wang et al., 2022
		Genome	Zhao et al., 2025
Megaloptera	JAK/STAT, Toll, IMD	Genome	Zou et al., 2024
		Transcriptome	Yang et al., 2024
Strepsiptera	JAK/STAT, Toll, IMD	Genome	Castaño et al., 2024
Mecoptera	JAK/STAT, Toll, IMD	Genome and transcriptome	Lasne et al., 2023
		Genome	Sivell et al., 2024
Trichoptera	JAK/STAT, IMD	Genome	Coleman et al., 2024
		Genome	Ge et al., 2022
		Genome	Luo et al., 2018
